# Relationship between rickets and incomplete distal renal tubular acidosis in children

**DOI:** 10.1186/1824-7288-36-54

**Published:** 2010-08-11

**Authors:** Abiola O Oduwole, Olayiwola S Giwa, Rasheed A Arogundade

**Affiliations:** 1 Department of Paediatrics, Lagos University Teaching Hospital, Idi-Araba, Lagos, Nigeria; 2 Department of Surgery, Lagos University Teaching Hospital, Idi-Araba, Lagos, Nigeria; 3 Department of Radiodiagnosis and Radiotherapy, Lagos University Teaching Hospital, Idi-Araba, Lagos, Nigeria

## Abstract

**Background:**

In the Sub Saharan Africa Rickets has now been established to be due primarily to calcium deficiency and sometimes in combination with vitamin D deficiency. The main thrust of management is calcium supplementation with or without vitamin D. An observation was made that some children with nutritional rickets do not respond to this management modality. The recently reported high prevalence of Incomplete Distal Renal Tubular Acidosis (idRTA) in adults with osteoporosis as brought to fore the possibility of this being a possible cause of calcium wastage and therefore the poor response in these group of children with rickets.

**Aim:**

To determine the prevalence of idRTA amongst a cohort of subjects with rickets

To show a relationship between rickets and incomplete distal renal acidosis

To determine the response of children with rickets and idRTA to addition of Shohl's solution to therapy

**Methodology:**

Two separate cohorts of children with rickets performed the ammonium chloride loading test to detect those with incomplete renal tubular acidosis. Following identification for idRTA, Shohl's solution was added to therapy of calcium and vitamin D supplementation and their response compared to those without idRTA on calcium and vitamin D supplementation solely.

**Results:**

50 children with rickets aged from two to six years of age and composed of 29 females and 21males were investigated. Incomplete renal tubular acidosis was found in 38% of them. Prevalence of idRTA was highest amongst those aged 3-6 years of age. Those with idRTA had worse limb deformities, biochemical and radiological parameters than those who hadn't. Rate of response on those with idRTA treated with Shohl's solution was at par with those without idRTA.

**Conclusion:**

Incomplete idRTA exist amongst children with rickets and should be looked out for in severe rickets and older children. Treatment of idRTA will lead to optimal response and healing of rickets.

## Background

Rickets a disease of deficiency is still a common finding in the developing countries amongst the under 5 children. Consequent to report from studies on rickets in children from the developing countries it was accepted that calcium deficiency and very occasionally vitamin D deficiency was the main basis for its development[1-8]. Although rickets peak age of presentation is between the age 1- 3 years [1-3], there has been a disturbing observation of rickets in children 5 years and older. It is being contemplated that the basis of rickets in these older children may be through another mechanism or the calcium deficiency which was also observed was not purely a nutritional deficiency [1-8].

Renal tubular acidosis has been known to be associated with rickets and the recent reporting of high prevalence of incomplete distal renal tubular acidosis (idRTA) in adults with osteoporosis [9], has brought into focus the effect of idRTA on skeletal parameters and the possibility of a similar effect in children.

Incomplete distal renal tubular acidosis (idRTA) is a type of primary distal renal tubular acidosis type 1, in which the individual is unable to acidify urine. The impaired distal acidification is characterized by an inability to lower urinary pH maximally (< 5.5) under the stimulus of systemic acidemia but because of due to a compensatory high rate NH_4_^+ ^excretion for the limited amount of titrable acid there is no associated severe metabolic acidosis[10,11].

Even though, unlike typical primary distal ubular acidosis, the metabolic acidosis in idRTA is mild, its chronic nature and the high urine pH initiate loss of calcium to into urine. This loss of calcium in the urine invariably leads to compensatory mechanisms in, which the bone is dissolved to release calcium though at a slower rate than in the typical RTA into the body system. Unfortunately the released calcium is lost through the kidneys into the highly alkaline urine perpetuating the hypocalcaemia, more leeching of the bone, further softening of the bone and subsequent rickets in a growing child. Other issues such as an associated abnormal vitamin D metabolism at the renal tubular level or calcium nutritional deficiency may accentuate the effect of the idRTA. A secondary hyperparathyroidism state usually arises from a combination of these events. Hyperparathyroidism has a direct effect on the tubules by increasing the rate of excretion of bicarbonate and invariably increasing the loss of mineral salt into the urine [10-12].

Diagnosis of Incomplete renal distal tubular acidosis is made when there is a demonstrable inability to lower urine pH below 5.5, either after NH_4_Cl loading or after furosemide administration. To correct this chronic alkalinization of the urine buffering solution such as Shohl's solution is given. After ingestion it is metabolized into bicarbonates. The bicarbonates released increases the urinary pH by increasing its excretion as free bicarbonate ions without producing systemic alkalosis. By this action it reverses the loss of calcium into urine into reabsorbing thereby increasing blood calcium level.

However, the following condition must be ruled out before a diagnosis of incomplete renal acidosis is made: urinary tract infections, low potassium levels, and sodium retention. Urinary tract infections can raise the pH level of the urine. Low levels of potassium can cause increased ammonia production, which will then lead to increased urine pH since ammonia is an alkaline substance [10].

We are hypothesizing that incomplete renal tubular acidosis may be a causal mechanism for rickets amongst children seen with rickets and a cause for poor response to therapy.

## Subjects and Methods

The study design is a prospective observational study undertaken at the Lagos University Teaching Hospital which ran from April to December 20008 after institutional ethics review board committee approval.

### Subjects Selection

Two cohorts of children were investigated. The first sets of cohort (cohort A) were five children who had been placed on calcium supplementation and vitamin D (cholecalciferol) in appropriate dosage for six months without an appreciable improvement in their biochemical and clinical status. The second set of cohorts (cohort B) were fifty two consecutively presenting children with rickets, who were older than 2 years referred to the paediatrics endocrine outpatient for limb deformity. Any child that was included in the study conformed to the inclusion and exclusion criteria as stated below.

### Inclusion Criteria

#### Clinical Features for diagnosis of rickets

Subject must have limb deformities with evidence of rachitic changes in the epiphyses, such as widening and knobby prominence of the wrist and ankle joints and rachitic rosary. These are common clinical features seen in Nigerian children with rickets according to findings by Thatcher et al [5,7] and Oduwole et al [13].

Apart from the above criteria, subjects that are included into cohort A must also not be responding to calcium supplementation and cholecalciferol therapy at six months assessment of clinical and biochemical features post institution of therapy. Such a subject was deemed not responding to therapy.

Physical examination was performed by the principal investigator for all the children.

#### Biochemical Indices for diagnosis of rickets

Subject must have the following biochemical parameters, low serum calcium, serum phosphorus which may be low, or normal and high serum alkaline phosphatase. Interpretation of biochemical values were based on the laboratory normal range values for age. The laboratory normal ranges were serum calcium 2.2-2.8 mmol/L, serum phosphate 0.7-1.3 mmol/L and serum alkaline phosphatase 40-90 U/L. All laboratory indices were done in the tertiary laboratory using their quality control method.

Subject in cohort A must also at six months assessment of biochemical features post institution of therapy have continuous lowering of serum calcium and phosphate levels and increasing level of plasma alkaline phosphatase.

#### Radiological Indices for diagnosis of rickets

The radiological survey of the limbs must show evidence of changes in the epiphyses such as osteopenia, widening of growth plate, decrease radio-density at sub-zone of provisional ossification.

Subject in cohort A, must also show depreciation in their radiological skeletal survey after being on calcium supplementation and cholecalciferol therapy for minimum of six months.

The radiology reports were made by the same senior radiologist to provide standardization, prevent error and bias.

### Exclusion Criteria

Any subject who had the following was excluded. These are hemoglobinopathy, urinary tract infection, evidence of hypokalemia or sodium retention to rule out hypoaldosteronism. Other exclusion criteria were severe protein malnutrition, liver disease, chronic diarrhea or malabsorbtion, abnormal serum creatinine level and any child whose caretaker consent could not be obtained. Appropriate laboratory investigations were done to rule out these parameters. Children on drugs that could be associated with rickets like phenobarbitone were also excluded. Any child below the age of 2 years was excluded because of the long hours of the test.

A total of seven subjects were excluded for either non participation in the ammonium chloride test or lack of parental consent. One was excluded from cohort a group and six from the cohort B group. The remaining 50 children studied filled a preformatted tested questionnaire to obtain biodata, type of housing, average daily period of exposure to sunlight, three days food diary, drug history and parents level of education and income to classify into social economic class using the Olusanya et al criteria [14].

### Urine calcium Measurement

24 hours urine calcium evaluation was done for each subject. The children were ambulatory and on a free diet. Prior to the day of the ammonium chloride test the caretakers or parent were advised to discard the first urine passed on waking up on the day of collection, subsequently all urine passed that day and that passed first thing on the day of test was collected into a specific container and brought to the test area. Urine collected were not exposed to sunlight or kept in a hot environment. Where a refrigerator was available, urine were kept in it or in a cool place in a black bag. Volume of urine collected was measured and then evaluated for its urine calcium concentration. Calciuria level in relation to the body weight have been found to be relatively constant in children except for a decrease during puberty [15]. Using the body weight, subject was deemed hypercalciuric if 24 hours urine calcium was more than 1.0 mmol/kg/day (4 mg/kg/day) [15,16]. This cut off point was used by Manz et al [15] during their study of 24 hour urinary calcium in healthy British and African children and it was found dependable.

### The short time ammonium chloride test

The subjects performed the modified short term ammonium chloride loading test after conforming to the inclusion and exclusion criteria and properly filling the preformatted form. The short term ammonium chloride test which is the modified form of the loading test by Wong and Davies [17] lasting for eight hours was chosen because it has been found to be sensitive and tolerated by children[18-20].

The children collected their urine for 24 hours prior to coming for the loading test as stated above. On arrival at the testing room, child was made comfortable. The subjects' last meal was at least eight hours prior to test. Test commenced from 7 am and ended at 1 pm. On arrival fresh urine and blood samples were collected for baseline urine pH and serum bicarbonate. This was followed by ingestion of ammonium chloride solution calculated at 0.1 gm/kg and dissolved in 100 mls of water. Ammonium chloride is a colorless and odorless type of salt that should make the blood slightly more acidic when introduced. Thereafter urine was collected every 2 hours over the next eight hours.

Urine pH was determined immediately after voiding or collection to prevent loss of carbon dioxide into the atmosphere. Blood samples were collected twice during the study, at the beginning of test and 3 hours after ingestion of solution. The blood samples were analyzed within a maximum of the hour of collection to prevent loss of CO2 which will give an incorrect (lower) value of bicarbonate. Electrolytes namely sodium, chloride and potassium were also measured. This was to calculate the anion gap thereby differentiating the type of acidosis present in the subject. The urine pH was analyzed in duplicate using the pH meter Orion Research Ionalyser/model 399A, Cambridge, Mass, USA, after standardization with different known buffer solutions provided with the pH meter. A child was said to be unable to acidify the urine if the pH remained above 5.5 after eight hours of ingestion of ammonium chloride solution. The normal serum bicarbonate concentration range is 22-30 mmol/L. In cases of tubular acidosis bicarbonate lower than 20 mmol/L may signify acidosis though mild and must be without any abnormal anion gap [21]. Therefore a fall of serum bicarbonate to 18- 20 mmol/L without any abnormal anion gap was used as a cut off point for classification of mild acidosis.

#### Management therapy

There were two treatment modalities.

### Modality 1 for those without idRTA

Subjects were placed on 5000 I.U of vitamin D in Arachis oil (calciferol) daily orally and one tablet of effervescence CaC1000 by Sandoz to give 600 mg of elemental calcium daily. Dietary counseling was given on calcium rich foods and calcium fortified foods or drinks such as fish bone, milk, egg, butter/margarine, cocoa drinks to augment the supplement from CaC 1000 by Sandoz.

### Modality 2 for those with idRTA

Shohl's solution was added for alkalization of the urine to modality 1. Shohl's solution is a palatable solution, which is a combination of sodium citrate and citric acid. It is readily metabolized into bicarbonate and gives an equivalent of 1 ml = 1 mmol of bicarbonate. It was given at a dose of 2 mmol per kilogram of body weight per day in three divided doses for easy timing and compliance by subjects. It is eliminated via urine with less than 5% unchanged. Shohl's solution was continued for all subjects with idRTA after healing had been achieved. It has minimal side effect such has nausea, vomiting, stomach pain and water retention from sodium in the sodium citrate [22].

Any subject in cohort A without idRTA would be managed as a case of vitamin D resistant rickets.

#### Follow-up

Subjects were seen at six weeks interval in the outpatient. On presentation subjects were examined by the investigator for changes in clinical features of rickets, any sign of sodium retention such as rapid weight gain, blood pressure measurement. Blood was taken to monitor changes in the biochemical indices. Radiological survey was repeated after six months of therapy modality. The values of the pre and post introduction of Shohl's solution to the therapy parameters were compared. Any improvement or otherwise was noted.

### Statistical analysis

Statistical analysis was done using the Microsoft excels software. Results are expressed as mean (SD), (%), z score, student t-test, 95% CI, probability (p <0.01).

The study protocol was approved by the ethical review committee at Lagos University Teaching Hospital and consent forms were signed by the caretakers of the subjects. There were no conflicting interests.

## Results

Fifty (50) subjects with rickets were studied after exclusion of seven from the 57 subjects from cohort A and B. They consisted of twenty one males and twenty nine females at a ratio of 1:1.3. Their age ranged from 2 to 7 years. Of these children 40 (80.00%) were between the ages 2 to 3.99 years and 10(20.00%) were between the ages 4 to 7 years. See table 1.

**Table 1:** Demographic characteristics of 50 studied children

	n (%)	Z value at 95% CI, p
**Gender**		
Female	29 (58)	1.4, p < 0.05
Male	21(42)	
		
**Age**		
2- 2.99 yrs	16 (32)	
3 - 3.99 yrs	24 (48)	
4 - 4.99 yrs	6 (12)	
5 - 5.99 yrs	3 (6)	
6 - 6.99 yrs	1 (2)	
Total no of subjects 2 -3.99 years	40 (80)	5.8, p < 0.05
Total no of subjects 4 -6.99 years	10 (20)	
**Socio-economic Class**		
Low socio-economic	40 (80)	5.8,p < 0.05
Mid socio-economic	10 (20)	
**Weight**		
> 80% expected weight	20 (40)	1.8, p < 0.05
< 80% expected weight	30 (60)	
% iDRTA <80% expected weight	16 (84.21)	2.327, p < 0.05
% non iDRTA <80% expected weight	14 (46.67)	
**Baseline Biochemical Parameters**		
Serum Alkaline Phosphatase (U/L)		
Mean (SD)	798 (82.84)	
Range	206-1,430	
Serum Calcium(mmol/L)		
Mean (SD)	1.92(0.08)	
Range	1.20-2.23	
Serum Phosphate (mmol/L)		
Mean (SD)	1.13 (0.08)	
Range	0.56-2.10	

Review of the three day recount food diary showed that at least 75% of the subjects had pap (corn meal) or tea with milk mixture every morning in combination with other foods. The amount of milk (powdered) used varied amongst the cohort from a teaspoonful to 2 tablespoons scoops of milk. Egg boiled or fried was seldom taken, either once or none per week. Fish (Tilapia or sardine or mackerel) was eaten daily but portion was small (a cultural belief that giving a bigger portion will egg on the child to steal encouraged this practice). Shrimps alone were not a common part of the diet usually a small portion may be added to vegetable soup. Liver was eaten by about 15% of cohort occasionally. A pat of margarine with bread was a common food item eaten by 60% of subjects. None took whole milk drink. Though the actual calcium intake could not be accurately assessed, it was obvious from the food portion size and frequency of intake that the daily calcium intake was not optimal.

19 (38.00%) of the subjects were unable to lower their urine pH below 5.5 after 8 hours of ingesting 0.1 mg/kg of ammonium chloride dissolved in water. They were considered to have incomplete distal Renal Tubular Acidosis (idRTA). The urine pH range was 5.7 to 7.50 with a mean of 6.40(0.09). 31(62.00%) could lower their urine pH below 5.5 and had a urine pH range of 4.62 to 5.48 with a mean of 5.10(0.07) There was a statistical difference *p*. <0.001 when the two groups urinary pH were compared. See table 2.

**Table 2:** Ammonium chloride loading test profile

	urine pH > 5.5	urine pH < 5.5	2t-test at 95% CI (p)
n (%)	19 (38.00%)	31(62.00%)	
Urine pH, mean (SD) range	6.40 (0.45) 5.7 -7.50	5.10 (0.32) 4.62 - 5.48	11.9275(< 0.001)
Preloading mean(SD) and range of serum HCO3,	19 (0.02), 19 - 20 mmol/L	22 0(0.461)., 22-24 mmol/L.	28.2313 (< 0.001)
3 hours post loading with NH4CL mean(SD) and range of serum HCO3	16 (0.34), 14 - 16 mmol/L	22 (0.58), 21-23 mmol/L	40.893 (< 0.001)
Rate of reduction of HCO3 from pre loading to 3 hours post loading	3-5 mmol/L	1-2 mmol/L	
24 hours urine calcium mean (SD)	1.62 (0.13)mmol/kg/day	0.12 (0.03)mmol/kg/day	20.4594 (< 0.001)

Of the 19 patients who were unable to lower their urine pH below 5.5, 13(68.42%) were aged between 3 to 6 years. Of these 13 subjects 8 (75%) were aged between 4 to 6 years. The mean urine 24 hour calcium was 0.12 mmol/kg/day for those able to lower their urine pH below 5.5, while those unable to lower their urine pH below 5.5, it was1.62 mmol/kg/day. See table 2. The 24 hour urine calcium value of 1.47-1.83 mmol/kg/day was observed for the three subjects with the highest urine pH value.

The preloading serum HCO3 observed for those whose pH were more than 5.5 was between 19 to 20 mmol/L while those whose pH were less than 5.5 had a serum HCO3 of 22-24 mmol/L. At 3 hours post loading with NH4CL, those whose urine pH was more than 5.5 had a reduction of serum HCO3 by 3-5 mmol/L to a new range of 14 to 16 mmol/L. see table 2. The lowest starting serum HCO3 of 19 mmol/L and highest reduction to 14 mmol/L at 3 hours post loading was observed in the two subjects with the highest urine pH of 7.01-7.50. For those whose pH was less than 5.5, 75% of them at three hours post loading maintained their initial serum HCO3 concentration. The remaining 25% had only a point reduction from their initial level of serum HCO3 concentration. Comparison of the two urine pH status was significant p < 0.001. The calculated anion gap for all the subjects who had mild acidosis was within normal range 8-12 mmol/L.

The mean plasma alkaline phosphate 798(82.84)U/L for those whose pH were more than 5.5 was observed to be higher than those whose pH were less than 5.5, 679 (64.60)U/L. This was statistical significant *p < 0.001*. The highest plasma alkaline phosphatase value of more than 700 U/L was recorded for the two subjects with the highest urine pH. See table 3. For both groups of subjects irrespective of their response to the loading test more than 90% of them had hypocalcaemia. But for the serum phosphate concentration the reversal was observed as more than 90% had normal levels. The mean values for these parameters corresponded to this observed trend (see table 3).

**Table 3:** Comparison of follow up outcomes of cohort B subset after commencement of appropriate modality

	urine pH > 5.5 (idRTA)				urine pH < 5.5				p
	Baseline	3 months	6 months	9 months	Baseline	3 months	6 months	9 months	
serum alkaline phosphate (U/L) mean (SD)	798 (82.84)	617 (55.67)	533 (55.02)	375 (53.21)	679 (64.60)	489 (51.40)	447 (60.00)	299 (46.82)	P < 0.01
serum calcium (mmol/L) mean (SD)	1.92 (0.08)	2.01 (0.06)	2.12 (0.03)	2.25 (0.5)	1.98 (0.07)	2.13 (0.08)	2.20 (0.080	2.28 (0.08)	P < 0.01
serum phosphate (mmol/L) mean (SD)	1.13 (0.08)	1.26 (0.07)	1.47 (0.06)	1.50 (0.06)	1.10 (0.09)	1.24 (0.08)	1.54 (0.07)	1.59 (0.05)	P < 0.01
Mean Height range (cm)	72.00 to 83.00				74.00 to 91.00				
Mean height velocity range (cm)	4.50 -8.00				4.00 - 8.40				

An observation also made was that the two patients with the windswept leg deformity had the highest urine pH between 7-7.50. Certain characteristic physical features of rickets were also observed to be more common and severer amongst those who were unable to lower their urine pH see table 4.

**Table 4:** Percentage occurrence of clinical features in the two subgroups

	Urine pH above 5.5 (19, 38%)	Urine pH below 5.5 (31, 62%)	Z value at 95% CI, p
Limb deformity	19 (100)	31 (100)	
Widening of the wrist and ankle joint,	19 (100)	31 (100)	
Windswept deformity	4 (21)	0 (0)	1.553, p > 0.05
Pain on walking	17 (90)	14 (46)	2.883, p < 0.05
Bossing of the skull	16 (84)	21 (67)	0.957, p > 0.05
Rachitic rosary	6 (31.8)	4(13)	1.239, p > 0.05
Chest deformity	5 (26.3)	5 (16)	0.51, p > 0.05
Delayed closure anterior fontanel	3 (15.8)	4 (13)	-0.134, p > 0.05
Poor dental status (caries, loss of teeth, malocculusion etc)	3 (15.8)	0 (0)	1.052, p > 0.05
Tetany/convulsion	1 (5)	0 (0)	

At follow up the subject in cohort A who hitherto had not improved on previous management showed a remarkable improvement in all parameters including weight and height see table 5.

**Table 5:** Profile of cohort a subjects with idRTA

	Pre Ammonium chloride loading test			Post Ammonium chloride loading test and intervention with shohls solution		
	Diagnosis	3 months	6 months	3 months	6 months	p
**Mean age (yrs)**	4.95 ± 0.36					
**Mean wt (kg)**	7.62					
**Mean serum calcium (mmol/L)**	2.15	1.90	1.80	2.37	2.48	P < 0.01
**Mean serum phosphate (mmol/L)**	1.36	1.46	1.49	1.42	1.21	P < 0.01
**Mean plasma alkaline phosphatase (U/L)**	925	1250	1234	810	660	P < 0.01
**Mean plasma bicarbonate** **(mmol/L)**	20	19	19	23	24	P < 0.01
**Radiography**	Florid rickets		No improvement		Healing Zone of calcification seen.	

For the subjects in cohort B, (see table 3) improvements in all parameters were at par in both subsets of subjects despite the idRTA and by six months all parameters were at their normal ranges. Improvements of clinical features of rickets were also at par in both subset of cohort B.

Unfortunately about 3 of the subjects stopped their use of Shohls solution after healing of rickets was obtained. The solution was stopped by their parents unilaterally despite counseling and they were ultimately lost to follow up. Another four were using their solution but erratically after healing had been obtained.

## Discussion

Nutritional rickets has been associated with reduced calcium intake both in children and even adolescents [2-9]. The study done by Thacher et al [23] that looked analytically at the calcium intake of children with rickets found that dietary calcium intake was low amongst their Nigerian cohort. This observation and the constant finding of hypocalcaemia and not vitamin D deficiency amongst children from other studies [2-9], has given credence to the role of nutritional calcium deficiency in the aetiolgy of rickets. Disturbance of renal regulation of acid-base metabolism causing bicarbonate wasting, alkaline urine and mild acidosis has been observed amongst children with rickets from as far back as in the 1920 s by Burghess et al [24]. What was not stated categorically was if it was another aetiology of rickets in these children. Mankin et al [21] in his study of cohorts with rickets, osteomalacia and renal osteodystrophy observed amongst them bicarbonate wasting and found that unless alkalizing solution was added to therapy large doses of calciferol did not correct their rickets. The recently reported high prevalence of idRTA in adults with osteoporosis made Sharma et al [9] to check similar effect on the height of children. Although these children did not have rickets, they found that children with idRTA were shorter than those without and came to the conclusion that it may have a significant deleterious effect on their growth (height).

idRTA is an entity whose laboratory features is similar to what was observed by Burghess [24] amongst his cohort of children with rickets. Although presence of idRTA was not studied in these children the outcome if it had been done is better imagined. From the present study 38% can be assumed to have idRTA. Those who had the highest urine pH were those with the worst clinical features. They also had the highest urine calcium loss and the lowest serum calcium.

Hypocalcaemia due to nutrition deficiency has been accepted as the most common cause of rickets in the developing tropical country, as vitamin D deficiency rickets is seldom found amongst the children that had been studied [1-8]. More than 90% of the subjects had hypocalcaemia, this was expected taking into consideration their low dietary calcium intake Hypocalcaemia causes secondary hyperparathyroidism which promotes leaching of calcium from the bone and increase loss of calcium from the kidney. The minimal loss of calcium in the urine by the subjects without idRTA despite their calcium deficiency may due to this secondary parathyroid action on the tubule. This action of the parathyroid hormone in combination with the effect of idRTA may have led to the much lower serum calcium, hypercalciuria and subsequently more severe clinical features in the subjects with idRTA. The observation that at urine pH greater than 6.50 clinical features of severe rickets such as windswept deformity, chest deformity, loss of deciduous teeth and tetany became more commonly seen suggested that the degree of severity of idRTA is directly related to that of rickets. Relationship between idRTA and rickets can be further established by the observed response of the subject in cohort A with idRTA to addition of Shols solution to their initial modality 1 therapy. The good response of the subjects in cohort B with idRTA in comparison with those without also reinforced the association of idRTA and rickets. Mankin et al [21] also observed this amongst his cohort.

An important observation made, was the history of rickets amongst the family members of some of the patients in those with severe rickets and idRTA. The approved protocol did not include testing the family members therefore only few of the family members who were willing to perform the loading test did it. It was interesting to find two of the fathers had idRTA with mild bowing of their legs. This raised the possibility of a genetic component to this phenomenon which should be further studied to see if a familial tendency can be established.

The peak age for rickets is 1-3 years and rickets at an older age has been rare therefore its increasing occurrence needs more study. Oduwole et al from the preliminary report from their yet to be published work observed the presence of Vitamin D insufficiency amongst older children and adolescent. A child with vitamin D insufficiency, nutritional calcium deficiency and idRTA, will be more vulnerable to developing rickets. This is an association that requires further study.

The Ammonium chloride loading test is a test that is well tolerated and can be performed easily by a child. Although more studies is needed before idRTA can be associated with development of rickets an advocacy for the performance of this test in conjunction with other investigations performed to identify cause and type of rickets especially by older children with rickets is hereby advanced. Management of children with idRTA by Shol's solution is simple with minimal adverse effect. From the response of the patients, early addition to therapy in those with idRTA hastens improvement and healing. Unrecognized urinary infection is a common cause of morbidity amongst children it must always be tested for. A high urine pH may be secondary to urinary infection with urea-splitting organisms.

## Conclusion

Rickets is still very much around and now unexpectedly, being seen in older children. It must be noted that the children with the worst clinical, biochemical and radiological features of rickets and the highest value of urine pH were the older children. Our Cohort showed that idRTA may exist amongst children with rickets and may be worth looking out for especially in older aged children, those with severe clinical features and poor response to therapy.

## Competing interests

'The authors declare that the manuscript has been done without any financial support and that they have no competing interests

## Authors' contributions

All the three authors have taken full responsibility for the paper and have read and approved its submission. AOO contributed to the conception, designing, acquisition of data, analysis and interpretation of data, drafting and critical revision for important intellectual content. OSG contributed to design, acquisition of data, and interpretation of data, critical revision for important intellectual content. ARA contributed to design, acquisition of data and critical revision for important intellectual content.

## Authors information

AOO is a Peadiatric endocrinologist at the College of Medicine, University of Lagos and Lagos University Teaching Hospital, Lagos. OSG is an Orthopaedic surgeon, at the College of Medicine, University of Lagos and Lagos University Teaching Hospital. Lagos. ARA is a Radiologist at College of Medicine, University of Lagos and Lagos University Teaching Hospital, Lagos
